# Discovery and Validation of Predictive Biomarkers of Survival for Non-small Cell Lung Cancer Patients Undergoing Radical Radiotherapy: Two Proteins With Predictive Value

**DOI:** 10.1016/j.ebiom.2015.06.013

**Published:** 2015-06-19

**Authors:** Michael J. Walker, Cong Zhou, Alison Backen, Maria Pernemalm, Andrew J.K. Williamson, Lynsey J.C. Priest, Pek Koh, Corinne Faivre-Finn, Fiona H. Blackhall, Caroline Dive, Anthony D. Whetton

**Affiliations:** a Stoller Biomarker Discovery Centre, Manchester Academic Health Science Centre, The University of Manchester, Wolfson Molecular Imaging Centre, Manchester M20 3LJ, UK; b Clinical and Experimental Pharmacology Group, Cancer Research UK Manchester Institute, Manchester Academic Health Science Centre, Christie Hospital, University of Manchester, Manchester M20 4BX, UK; c Karolinska Institutet, Scilifelab, Department of Oncology and Pathology, Tomtebodavägen 23, 171 65 Stockholm, Sweden; d Faculty Institute of Cancer Sciences, Manchester Academic Health Sciences Centre, University of Manchester, M20 4BX, UK; e The Christie NHS Foundation Trust, Wilmslow Road, Manchester M20 4BX, UK

**Keywords:** AC, adenocarcinoma, CEA, carcinoembryonic antigen, CRP, C-reactive protein, EGFR, epidermal growth factor receptor, FDR, false discovery rate, IL-6, Interleukin 6, iTRAQ, isobaric tagging for relative and absolute quantification, LBP, lipopolysaccharide binding protein, LRG1, leucine-rich alpha-2-glycoprotein, mo, months, MS/MS, tandem mass spectrometry, NSCLC, non-small cell lung cancer, PCA, principal component analysis, SCLC, small cell lung cancer, SqCC, squamous cell carcinoma, TEAB, triethyl ammonium bicarbonate, VEGF, vascular endothelial growth factor, v/v, volume/volume, Lung cancer, Radiotherapy, Biomarker, Proteomics

## Abstract

Lung cancer is the most frequent cause of cancer-related death world-wide. Radiotherapy alone or in conjunction with chemotherapy is the standard treatment for locally advanced non-small cell lung cancer (NSCLC). Currently there is no predictive marker with clinical utility to guide treatment decisions in NSCLC patients undergoing radiotherapy. Identification of such markers would allow treatment options to be considered for more effective therapy. To enable the identification of appropriate protein biomarkers, plasma samples were collected from patients with non-small cell lung cancer before and during radiotherapy for longitudinal comparison following a protocol that carries sufficient power for effective discovery proteomics. Plasma samples from patients pre- and during radiotherapy who had survived > 18 mo were compared to the same time points from patients who survived < 14 mo using an 8 channel isobaric tagging tandem mass spectrometry discovery proteomics platform. Over 650 proteins were detected and relatively quantified. Proteins which showed a change during radiotherapy were selected for validation using an orthogonal antibody-based approach. Two of these proteins were verified in a separate patient cohort: values of CRP and LRG1 combined gave a highly significant indication of extended survival post one week of radiotherapy treatment.

## Highlights

• We demonstrate that the plasma proteome of lung squamous cell carcinoma patients are changed during radiotherapy.
• We demonstrate that using a combination LRG1 and CRP proteins patient survival can be predicted.

**Research in Context:**

Lung cancer is the most frequent cause of cancer-related death world-wide. The main curative treatment is surgery but due to diagnosis at a late stage or complicating factors this is not an option for the majority of patients, and so radiotherapy is common. Radiotherapy is known to cause severe toxicity which can be life threatening but there is no markers which can be used to guide treatment. Therefore we have studied the changes in blood during radiotherapy, identifying two proteins which predict survival. These proteins may allow radiotherapy of lung cancer patients to be guided early during treatment.

## 1 Introduction

In the era of personalised medicine, biomarkers are required for the stratification of patients allowing therapy to be tailored. This could include molecular histology of disease to allow driver mutation targeted therapy, for example EGFR tyrosine kinase inhibitors for lung cancer patients (Lynch et al., 2004, Paez et al., 2004, Pao et al., 2004). Biomarkers which can be used as early markers of response to treatment would be particularly useful in the clinic as well as in drug development, allowing patients therapy to be tailored as early as possible (Beretta, 2007). To be used routinely in the clinic, a biomarker would have to be measurable in a non-invasive readily accessible tissue or biofluid. Plasma as well as urine is routinely used in clinics for the diagnosis of a variety of diseases. For example, monitoring prostate specific antigen levels in blood has been used for screening and monitoring progression of prostate cancer (reviewed in Lilja et al., 2008).

A major issue for identification of protein biomarkers is the high dynamic range of protein content in plasma (of the order of 10^10^Polanski and Anderson, 2007) that can make mask lower abundance proteins reducing the opportunity for detection with current instrumentation. However advances in mass spectrometry and liquid chromatography coupled to the depletion of highly abundant proteins have allowed the plasma proteome to be investigated with approximately 6 orders of magnitude penetration allowing identification of so called tissue leakage proteins which are predicted to be rich in biomarkers (Rodriguez-Suarez and Whetton, 2013, Zhou et al., 2012). Another challenge of biomarker discovery is the large variation present both between individuals in a population and in an individual over time. We have previously published an analysis showing that with the appropriate use of longitudinal samples our isobaric tagging plasma proteomics workflow can be used to identify biomarkers from clinical studies with as few as three patients per group with a power of 0.8 for the 70% least variant proteins (Zhou et al., 2012). We have coupled this approach to our newly published bioinformatics technique which more accurately estimates specific protein technical variation, this additional modelling allows more proteins to be identified as differentially expressed with sufficient power (Zhou et al., 2013). To show the utility of these methodologies we have investigated if plasma markers with clinical utility can be identified in non-small cell lung cancer (NSCLC) patients undergoing radical radiotherapy in a deliberately small cohort (3 vs 3) using a longitudinal sampling approach. Two baseline samples prior to the start of radiotherapy were analysed from each patient, allowing the baseline variation of each protein to be assessed, and thus significant changes during radiotherapy identified. These changes were then validated in a second independent cohort of twenty three patients using a second methodology.

Patients diagnosed with lung cancer have a 5 year survival rate of < 10% in Britain (Parkin et al., 2005). Globally there are over 1.2 million deaths related to lung cancer per annum (Crino et al., 2010). Surgery remains the mainstay curative treatment for this disease. However the majority of patients present with disease that is too advanced to be resected or have multiple comorbidities precluding surgery. As a consequence radical radiotherapy, either alone or combined with chemotherapy, plays a major role in the treatment of patients with locally advanced lung cancer (Crino et al., 2010). Radiotherapy is known to cause acute and late toxicity in patients due to damage to surrounding normal tissue. An example of thoracic radiation toxicity is pneumonitis and pulmonary fibrosis which can be life threatening and can affect patients' quality of life and treatment outcome (reviewed in Abratt and Morgan, 2002). Therefore assessment of response to treatment such as radical radiotherapy is a valid and useful contribution to determining treatment options in those undergoing radical radiotherapy. We therefore considered if we can find predictive factors for survival after radical radiotherapy by use of our proteomics pipeline.

Here we report on the proteomic analysis of samples from the prospective study, RADAR, in which patients with small cell lung cancer or NSCLC who are treated with radical radiotherapy are asked to donate blood for research into toxicity and predicting outcome to treatment. The materials collected for this study were longitudinal in nature with samples taken prior to the radiotherapy and during treatment. This allowed us to look at proteins which can act as predictive markers of survival early in the radiotherapy treatment using global discovery proteomics. The results of this proteomic analysis are detailed below with potential markers identified and validated in an independent cohort.

## 2 Materials and Methods

### 2.1 Sample Collection

**Fig. 1:**
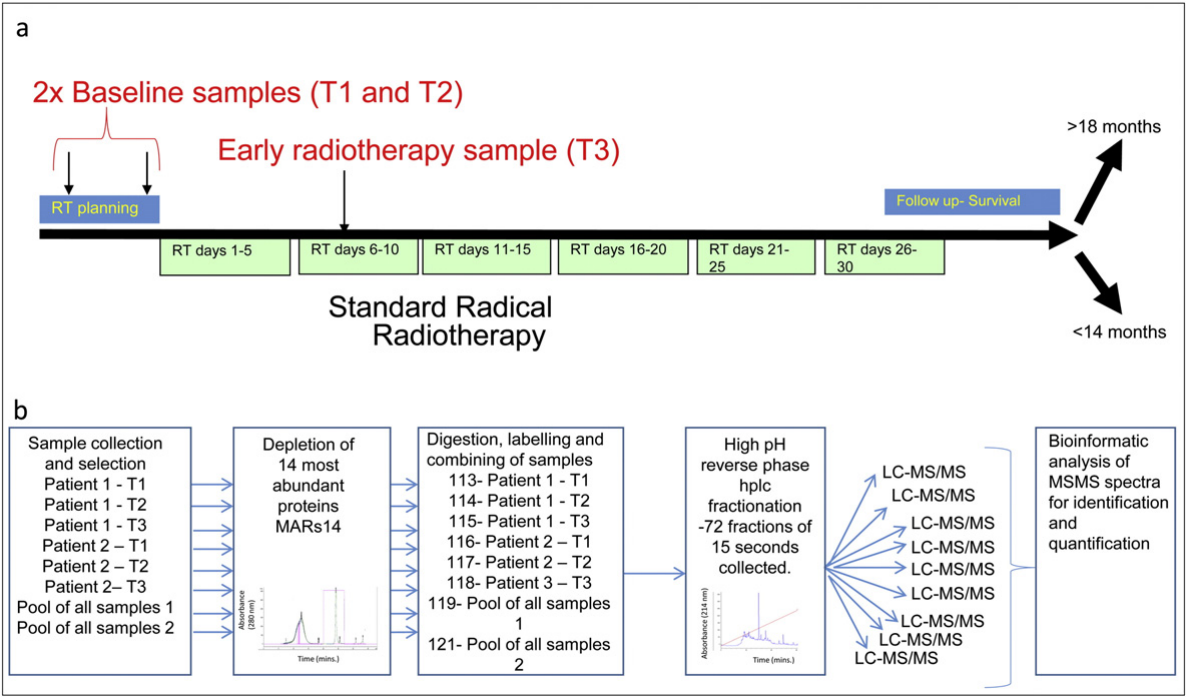
Schematic representation of study design and experimental workflow. (a) Therapeutic plan, two baseline samples are collected prior to the start of RT (T1 and T2). RT is delivered over 6 weeks (green boxes) with a sample collected during week 2 (T3). After treatment patient survival was followed and patients were retrospectively assigned to either the < 14 mo or > 18 mo survival groups. (b) Experimental plan and workflow for identification and relative quantification of plasma proteins. Proteins were depleted of high abundance proteins using a MARS 14 depletion system followed by digestion with trypsin and labelling with the correct iTRAQ reagent. Each iTRAQ 8 plex was designed to contain the samples from 2 patients (one < 14 mo one > 18 mo) A portion of all samples included in the discovery cohort were collected into a pool sample which was used to assess technical variation and allow comparisons across isobaric tagging experiments. Peptides were fractionated prior to mass spectrometry by 2-dimensional reverse phase liquid chromatography, the 1st dimension at pH 10.5 and the second at pH 3. The mass spectrometry was run with IDA methods and the raw result files analysed by Protein Pilot. Protein quantification is then reconstituted from high confidence peptide spectral matches and the proteins with elevated levels postradiotherapy identified.

Blood was collected from donors in lithium heparin coated tubes and centrifuged within 30 min of collection at 2500 ×*g* for 15 min at 4 °C before aliquots of the plasma layer were stored at − 80 °C. Samples were collected at the following time points for each patient; before RT, during RT (days 2, 3, 8, then weekly) and on completion after RT (months 1, 3, 6) (Fig. 1). Blood samples were taken from 29 randomised patients with lung cancer enrolled in the RADAR study at the Christie Hospital, Manchester, UK following written informed consent with ethical approval from the Central Manchester Local Research Ethics Committee. This proteomic analysis was undertaken on two samples per patient collected prior to the start of radiotherapy and a third sample on day 8 of the treatment regimen.

### 2.2 Proteomic Workflow and Experimental Setup

The experiment and workflow was carried out as in Fig. 1. A 50 μl aliquot of each sample in the study was pooled and used as a pooled internal control sample, analysed in duplicate in each isobaric tagging for relative and absolute quantification (iTRAQ) experiment to test technical variation. Each iTRAQ experiment consisted of two internal control samples (channels 119 and 121) and six samples from two patients randomised into the remaining channels. The study used three iTRAQ experiments to analyse samples from six patients.

### 2.3 Protein Depletion, Digestion and Labelling

Abundant proteins were removed from plasma using an Agilent Mars14 chromatography column following the manufacturers' protocol (Agilent, Palo Alto, CA, USA). Depleted samples were concentrated and exchanged into 1 M triethyl ammonium bicarbonate (TEAB) using 4 ml spin concentrators with a 5 KDa molecular weight cutoff filter (Agilent Palo Alto, CA, USA) as per manufacturer's instructions. The protein concentration in buffer-exchanged samples was measured using Bio-Rad protein assay reagent (Bio-Rad, Hercules, CA, USA). 50 μg of each sample was reduced with the addition of 1/10th of the sample volume of 50 mM tris(2-carboxyethyl)phosphine for 1 h at 60 °C. Cysteine residues were then alkylated by the addition of 1/20th of the total sample volume of 200 mM methyl thiomethanesulfonate (in isopropanol) before incubation for 10 min at room temperature. Protein was digested by the addition of 5 μg of porcine trypsin (Promega, Madison, WI, USA), followed by overnight incubation at 37 °C. The digested protein samples were isobarically tagged with 8plex iTRAQ reagents according to the manufacturers' instructions (ABSCIEX, Framingham, MA, USA). After labelling the samples were dried at 60 °C in a DNA concentrator (GeneVac, Ipswich, UK) and then stored at − 20 °C.

### 2.4 High pH Reverse Phase Chromatography

Isobarically tagged samples were reconstituted in 100 μl of buffer A (99.5% water adjusted to pH 10.5 with ammonium hydroxide) and appropriate samples pooled prior to being loaded onto a 100 mm × 4.6 mm 3 μm C18 HPLC columns (Agilent Palo Alto, CA, USA). Peptides were eluted by the application of a linear 30 min gradient up to 50% buffer B (Acetonitrile, 0.1% (v/v) ammonium hydroxide) with 70 × 15 s fractions collected from 4 min. Fractions were dried in a DNA concentrator (GeneVac Ipswich, UK) at 60 °C and stored at − 20 °C.

### 2.5 Mass Spectrometry (MS/MS)

Samples were reconstituted in 30 μl of samples loading buffer (20 mM citric acid, 2% (v/v) acetonitrile and 0.1% (v/v) formic acid). 3 μl of each fraction was then loaded onto a nanoACQUITY UPLC Symmetry C18 Trap, 5 μm, 180 μm × 20 mm (Waters, Milford, MA, USA) at 15 μl/min of 3% (v/v) acetonitrile, 0.1% (v/v) formic acid for 5 min. Analytical separation of the peptides was performed using nanoACQUITY UPLC BEH C18 Column, 1.7 μm, 75 μm × 250 mm (Waters, Milford, MA, USA). Briefly, peptides were separated over a 91 min solvent gradient from 3% (v/v) acetonitrile, 0.1% (v/v) formic acid to 40% (v/v) acetonitrile, 0.1% (v/v) formic acid. MS was carried out by a TripleTOF 5600 (ABSciex, Framingham, MA, USA) set up to analyse the top 20 ions by MS/MS per MS scan. The MS scanned between 350 and 1250 m/z with an accumulation time of 250 ms. Ions were only selected for MS/MS if they were over 150 counts per a second and had a charge state of between 2 and 5, ions previously selected were excluded for 30 s. The MS/MS was carried out in high sensitivity mode with 100 ms accumulation time and a rolling collision energy based upon mass and charge with a spread of 20. The MS/MS scanned between 100 and 1600 m/z.

### 2.6 Protein Identification

All MS/MS data were submitted to ProteinPilot software version 4.5 (ABSCIEX, Framingham, MA, USA) for database searching and iTRAQ reporter ion quantification. Searches were performed against the Ensembl human core 63 data base (85285 entries, downloaded 2012) with the following settings; cysteine alkylation with methanethiosulfonate (MMTS), biological modifications allowed and trypsin as the digestion enzyme. The search was carried out with default settings and thus the cleavage specificity, number of missed cleavages and mass tolerances were preset. The false discovery rate (FDR) of protein identification was controlled using a target-decoy searching strategy (Elias and Gygi, 2007) where forward and reverse sequences from a database were in equal competition to be the highest ranking identification for each spectrum. The q-value approach (Storey and Tibshirani, 2003) was then applied to define a cut-off for peptide confidence so that the control criteria of FDR can be met. The maximum allowed FDR for protein identification was set to 5%. The iTRAQ data has been deposited to the ProteomeXchange Consortium (Vizcaino et al., 2014) via the PRIDE partner repository with the dataset identifier PXD001052 and uploaded to http://www.scalpl.org/hank/MatchPage;jsessionid=80bf3253ea31befd6e36c2ce957a?0 under RADAR. Guide to the use of this database can be found in Supplementary Document 1.

### 2.7 Identifying Proteins That Are Differentially Expressed

Based on two baseline samples and one post-treatment sample per patient we aimed to discover proteins that were differentially expressed in response to radiotherapy, and in particular, to discover those that allowed discrimination between patients with good and poor survival. Aspects of data variation were investigated in order to assign reliable statistical significance to the observed protein changes.

Technical variation inherent in each iTRAQ experiment was estimated from the technical replicates included in the experiments, using the method as described in the supplementary material. This method allowed estimation of technical variation following a Normal distribution for each individual protein.

Within-person variation can be estimated by the changes between the two baseline samples. These changes were measured as log ratios and were considered to come from a combination of technical variation and within-person variation, therefore:

$$var\left(\log\frac{I_{2}}{I_{1}}\right)=var_{\mathrm{within}\;\mathrm{person}}+var_{\mathrm{technical}}$$

where *I*_1_ is the expression level at the first baseline and *I*_2_ is the expression level at the second baseline. Three assumptions had to be adopted to make the estimation of within-person variation valid:

1) There is no change in disease during the period from which baseline samples were taken.
2) Within-person variation didn't change in response to treatment, i.e., once the variation was estimated using the baseline data it can also be used in analysing the post-treatment data.
3) All proteins were assumed to follow a same Normal distribution. This assumption was adopted because two baseline samples didn't allow accurate estimation of within-person variation for each individual protein. The distribution of the baseline variation was illustrated in Supplementary Fig. 1 and the validity of the assumption is shown.
  All proteins were assumed to follow a same Normal distribution. This assumption was adopted because two baseline samples didn't allow accurate estimation of within-person variation for each individual protein. The distribution of the baseline variation was illustrated in Supplementary Fig. 1 and the validity of the assumption is shown.

The methodology described above was adapted from previous publications (Zhou et al., 2012, Zhou et al., 2013).

Inter-person variation was estimated as following a unique Normal distribution for each individual protein. If it was intended to find proteins that were differentially expressed following treatment, i.e. equal treatment effect were expected for all patients, inter-person variation should be estimated using all available patients. Otherwise, if it was intended to find proteins that change differently in patients from different cohort, inter-person variation had to be estimated separately for each cohort of patients. Inter-person variation was estimated by deducting technical variation and within-person variation from the observed variation within the defined cohort of patients, as published (Zhou et al., 2012).

With the technical variation, within-person variation and inter-person variation estimated for each protein, the statistical significance (p-values) of the observed changes on one protein can be readily defined. Proteins differentially expressed as a result of treatment were identified according to the p-values, and a q-value approach (Storey and Tibshirani, 2003) was applied for FDR control to ensure that only the most confident identifications were retained for further validation.

The acute phase response pathway diagrams were generated through the use of IPA (Ingenuity® Systems, www.ingenuity.com).

### 2.8 Verification of Potential Biomarkers

Levels of target proteins were measured using commercial ELISA kits, LRG1 (Demeditec, Germany), CRP (Invitrogen, Paisley, UK), LBP (Abnova, Taipei City, Taiwan) following manufacturer's instructions. IL6 was measured using the SearchLight Plus multiplex ELISA platform (Aushon BioSystems, Boston, US) and was run using the method previously described (Backen et al., 2009). The verification analysis was carried out with the sample outcome blinded until data analysis. Statistical analysis of the ELISA results was carried out using Graphpad Prism version 6.04 with significance testing done using unpaired two tailed Mann Whitney test.

## 3 Results

### 3.1 Discovery Proteomics Using Longitudinal Samples From NSCLC Patients Undergoing Radiotherapy

The analysis of plasma from patients and apparently healthy individuals for identification of potential biomarkers suffers from the heterogeneity of protein content in peripheral blood. We have shown that this can be overcome by use of longitudinal samples, a reproducible plasma preparation and isobaric tagging relative quantification mass spectrometry approach (Zhou et al., 2012).

The aim of the study was to identify proteins that significantly change during the first five fractions of a course of radiotherapy as candidates for biomarkers of clinical benefit from radiotherapy (Fig. 1a). Plasma was collected to a standard operating procedure (see Materials and Methods) from patients undergoing radical radiotherapy for lung cancer treatment. Patients were classified into two groups (n = 3) dependent on survival time: those living for < 14 mo after treatment (< 14 month group); those living > 18 mo after treatment (> 18 month group). The survival cut-offs were selected based on the median survival of the study being 17 mo. In both groups only squamous cell carcinoma patients with cancer related death were included and cases were balanced for clinical factors as far as possible (Supplementary Table 1). Two samples were collected prior to the start of radiotherapy enabling the “baseline” variation of the proteins in a specific patient to be assessed (see, Fig. 1a) and allowing smaller discovery cohorts of patients to be incorporated by design. The relative levels of proteins were measured using 8-plex isobaric tagging of peptides which allowed two patients to be analysed per MS run alongside a pooled reference, in duplicate. The pooled reference allowed technical variation to be observed, see Fig. 1b for proteomic workflow.

**Fig. 2:**
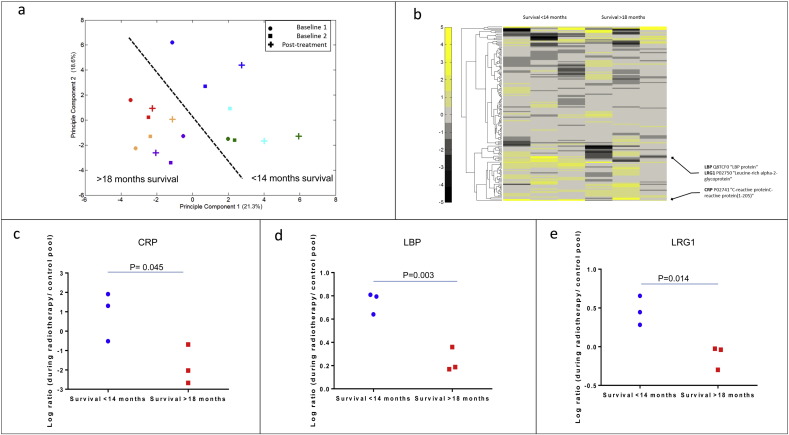
Discovery proteomics using longitudinal samples from NSCLC patients undergoing radiotherapy can distinguish patients with better survival. Patient samples were analysed using an isobaric tagging 2D LC MS/MS method described in the Materials and Methods. (a) The levels of all proteins relative to a pooled reference, quantified in all isobaric tagging mass spectrometry experiments (157 proteins) were analysed by unsupervised principal component analysis of all patient sample and control pools. The first and second principle components of the data were plotted in the figure with each data point an individual patient and time point. Colour represents an individual. Red, orange and purple survival > 18 mo, blue, green and cyan survival < 14 mo. The shape represents the time point; circle T1, square T2 and cross t3. (b) The log ratio of T3 to average of T1 and T2 of each protein for each patient was clustered. This cluster was visualised by plotting the data on a heat map with colour indicating the degree of difference calculated (black is reduced and yellow is increased). (c–e) Scatter plots of the three proteins (LRG1, CRP and LBP) changing significantly in the < 14 mo survival patients (blue circle) when compared to the > 18 mo survival (red square). All significance tests were two tailed unpaired t-tests.

Relative quantification on 658 proteins with a peptide FDR less the 0.05% on identification (Supplementary Table 3, Supplementary Table 4, Supplementary Table 5) was derived. The proteins identified were enriched for classical plasma protein pathways such as the complement cascade and acute phase response proteins as is standardly seen in such studies. The within and between person variation in plasma proteome during radiotherapy was investigated using unsupervised principal component analysis (PCA) of proteins quantified in all samples (Fig. 2a). By plotting the first and second principal component we showed that the largest variation in the dataset was between different patients; as was anticipated from our previous studies, validating our decision to use a workflow that only permits longitudinal analysis. This PCA also demonstrated that the two survival group (< 14 mo and > 18 mo) could be separated by their first two components, indicating that a proteomic biomarker could be used to discriminate between these patients. We observed that the largest intra-person variation after radiotherapy was observed in the three patients with survival < 14 mo (Fig. 2a). In order to investigate the plasma proteome dataset further we generated a heat map (Fig. 2b) taking the change during radiotherapy for each protein and patient. The clustering of protein did not show any pathways regulated in either patients group.

Potential protein biomarkers for radiotherapy prognosis were identified using the statistical approach described in the methods. With the technical and biological variation calculated for each protein, 9 putative biomarkers had a significant change during radiotherapy (Table 1). The list of significantly (p < 0.05) changing proteins was triaged further by manual inspection of spectra and data, proteins showing consistent changes across patients and low variation in the technical replicates of the pooled standards were prioritised. Three proteins with elevated levels during radiotherapy passed the manual inspection; C-reactive protein (CRP), Lipopolysaccharide binding protein (LBP) and Leucine-rich alpha-2-glycoprotein (LRG1). Fig. 2c, d, and e shows the ratio of iTRAQ protein concentration values using the pooled plasma reference standard as denominator for CRP, LBP and LRG1. Power analysis indicated that a power of more than 90% was reached, demonstrating that the selected protein had a sound statistical evidence.

### 3.2 Verification Analyses on Putative Biomarkers

**Fig. 3:**
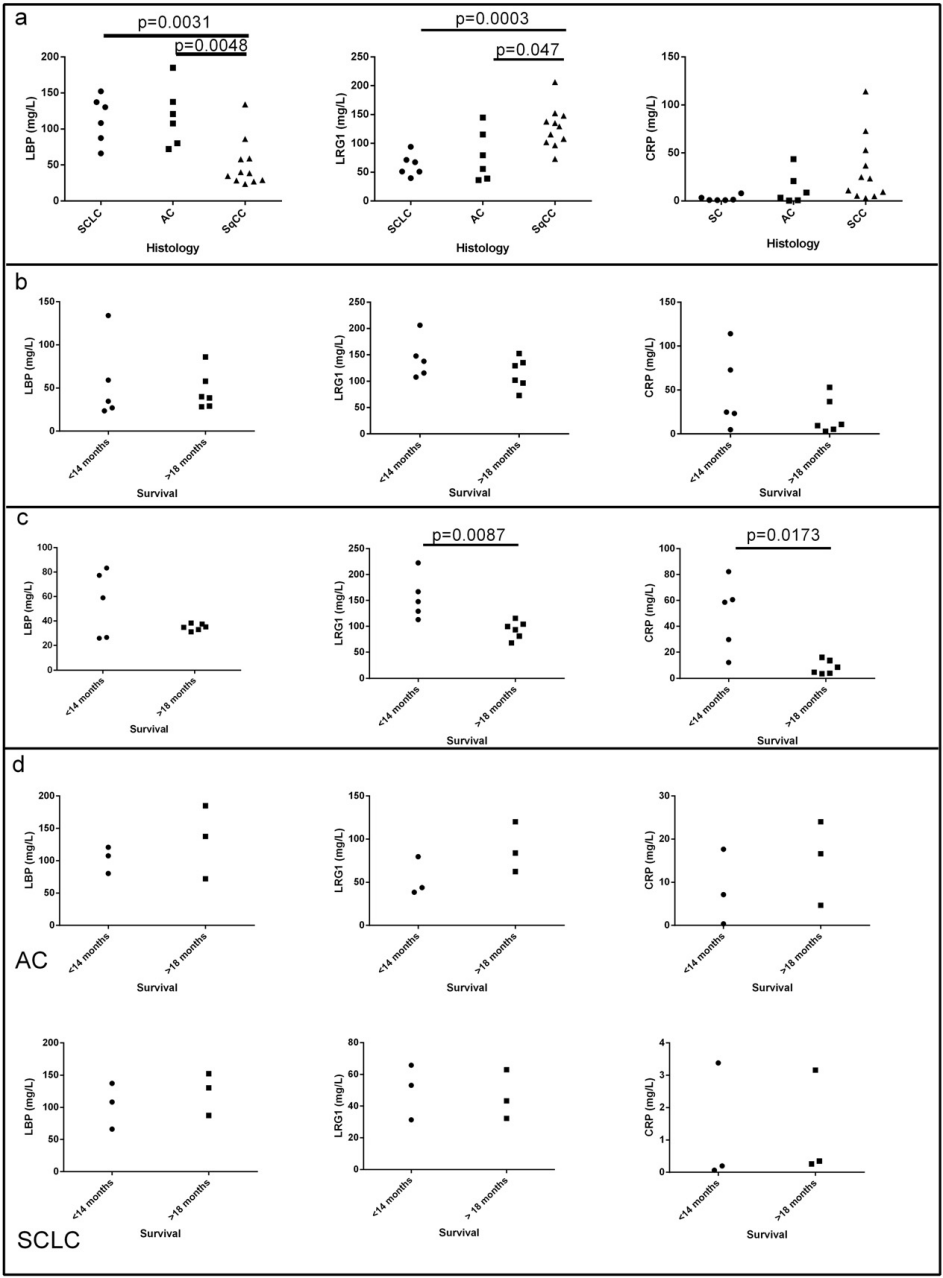
Verification of putative biomarkers in additional cohort and with orthogonal method. (a) Scatter plots of concentration of LBP, LRG1 and CRP in plasma with small cell lung cancer prior to radiotherapy (SCLC—circle), adenocarcinoma NSCLC (AC—square) or squamous cell carcinoma NSCLC (SqCC—triangle). Levels of the proteins in plasma were assayed by commercial ELISA. (b) Scatter plots of the concentration of LRG1, CRP and LBP in plasma from SqCC patients prior to radiotherapy comparing survival < 14 mo (circle) and > 18 mo (square). (c) Scatter plots of the concentration of LRG1, CRP and LBP in plasma from SqCC patients during radiotherapy comparing survival < 14 mo (circle) and > 18 mo (square). (d) Scatter plots of the concentration of LRG1, CRP and LBP in plasma from AC and SCLC during radiotherapy comparing survival < 14 mo (circle) and > 18 mo (square). Significance was tested using a 2-tailed unpaired Mann Whitney test.

To verify if the three shortlisted proteins are predictive of survival during radiotherapy an additional cohort of 23 additional lung cancer patients were analysed using enzyme linked immunosorbent assay (ELISA) for CRP, LBP and LRG1. In this further part of the study other lung cancer subtypes were included (Supplementary Table 3). Thus to test if the effect is only specific to squamous cell carcinoma NSCLC (SqCC), six patient samples from adenocarcinoma NSCLC (AC) and small cell lung cancer (SCLC) patients respectively were included in the verification samples set as well as eleven SqCC patients. Each patient had a single pre-treatment sample as well as an early radiotherapy treatment sample collected as for the SqCC NSCLC discovery proteomics sample set using the same standard operating procedures for sample collection and storage. The hypothesis that the putative biomarkers mentioned above had relevance in diseases other than SqCC NSCLC, like AC and SCLC, was tested by examining the level of each protein in patients' plasma prior to and after radiotherapy (Fig. 3a). CRP showed no significant difference between any patient groups prior to therapy. LRG1 levels in the plasma of SqCC patients (127 ± 11 mg/L mean ± SD N = 11) was significantly different to AC (78 ± 18 mg/L, mean ± SD N = 6 p < 0.047) and SCLC (62 ± 8 mg/L mean ± SD N = 6, p < 0.0003) prior to therapy. LBP levels in the plasma of SqCC patients (33 ± 11 mg/L mean ± SD N = 11) was significantly different to AC (13 ± 7 mg/L mean ± SD N = 6, p < 0.0048) and SCLC (3 ± 1 mg/L mean ± SD N = 6, p < 0.0031). Due to the differences seen in protein levels between the different patient sets the effect of radiotherapy on levels were only analysed between patients with the same histology. The levels of all three proteins (CRP, LBP and LRG1) prior to radiotherapy showed no significant difference in the plasma of patients with poor or good prognosis prior to radiotherapy (Fig. 3b). The level of each of the proteins was compared for their changes during radiotherapy and only SqCC patients showed any significant difference in protein levels between patients with good and poor prognosis (Fig. 3c). LBP was down regulated following radiotherapy, consistent with the MS/MS results, but it showed no significant difference between the > 18 mo survival SqCC patient group and the < 14 mo survival SqCC patient group. The level of CRP and LRG1 were both significantly different in the SqCC < 14 mo survival group (CRP 48 ± 27 mg/L, LRG1 156 ± 19 mg/L mean ± SD N = 5) compared to the SqCC > 18 mo survival group (CRP 8.5 ± 2 mg/L p = 0.0173, LRG1 94 ± 7 mg/L p = 0.0087, mean ± SD N = 6). Analysis of the levels seen before and during radiotherapy levels for LRG1, LBP and CRP in adenocarcinoma and small cell lung cancer showed no significant difference in values (Fig. 3d). A post-hoc power analysis of our two phase study (Mass spectrometry identification followed by ELISA verification) was carried out based on simulation, a power of 82.6% was achieved, indicating that our results are highly repeatable.

**Fig. 4:**
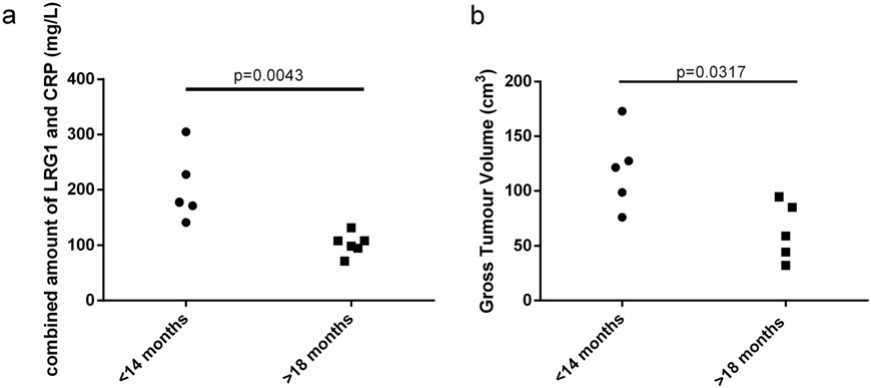
Combination of LRG1 and CRP as a multiplexed biomarker can discriminate between survival groups and may add value to the use of gross tumour volume as a predictor of survival. Scatter plots of survival less than 14 mo (circle) and more than 18 mo (squares) compared to (a) combined levels of CRP and LRG1 in the plasma of SqCC patients during radiotherapy and (b) gross tumour volume (cm^3^). Significance was tested using a 2-tailed unpaired Mann Whitney test.

Since both CRP and LRG1 were found to be significant predictive markers of survival for squamous cell carcinoma patients undergoing radiotherapy, we determined whether combining the levels of the two proteins linearly could be of value. This increased the significance of the difference between the good and poor prognosis groups (p = 0.0043). Using a cut-off value of a combined level of 140 mg/L all patients could be stratified into the correct group of < 14 mo and > 18 mo (Fig. 4a). LRG1 or CRP used in isolation does not result in this complete stratification. Gross tumour value was also available for ten of the patients (5 < 14 mo and 5 > 18 mo) analysed, since this has previously been shown to be a predictive marker of survival for radiotherapy of lung cancer patients this was also tested. This showed a significant difference (p = 0.0317) between the two survival groups but could not fully discriminate all the patients (Fig. 4b).

### 3.3 Acute Phase Response in Isolation is Unlikely to be Responsible for Elevation of the Putative Biomarkers LBP and CRP

**Fig. 5:**
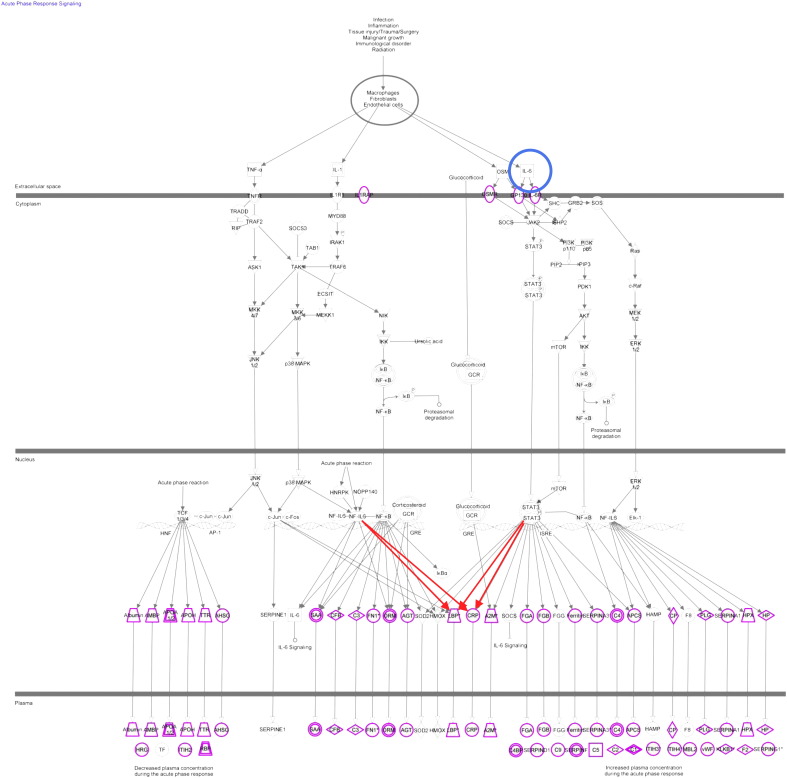
The acute phase response is not generally up regulated in patients with < 14 mo survival. The acute phase response pathway adapted from ingenuity software, highlighting the position of IL6 (blue circle) and the proteins identified in this study (purple) with up regulated proteins (red).

To understand the biological process that may cause or be related to the changes observed in < 14 mo survival patients in response to radiotherapy we analysed the contexts in which CRP and LBP may change. Ingenuity analysis showed that the acute phase response pathway is enriched in the identified dataset with 29 proteins from the pathway seen (see Fig. 5), however only CRP showed the expected difference between outcomes consistent with this pathway taking into account p value alone (Table 2). This indicates that the increased levels of CRP in < 14 mo survival patients may not be due to a general increase in the acute phase response. In the acute phase response CRP and LBP are both potentially regulated by changes in the levels of Interleukin 6 (IL-6) (Ganapathi et al., 1990, Castell et al., 1989, Castell et al., 1988, Moshage et al., 1988, Dentener et al., 2000, Grube et al., 1994). Furthermore a previous study identified IL-6 as a possible predictive marker for survival radiotherapy (Dehing-Oberije et al., 2011). The level of IL-6 was available for eight of the squamous cell carcinoma patients during treatment and so its correlation with CRP, LRG1 and LBP was investigated during radiotherapy. The correlation coefficient with IL-6 was significant for CRP with 0.86 (p value 0.006) and LRG1 0.751 (p value 0.032) but not for LBP with 0.52 (p value 0.191).

## 4 Discussion

In the treatment of any malignant disease with radiotherapy there is associated morbidity and mortality. The onset of next generation sequencing and other approaches in clinical medicine will in the future potentially enable the tailoring of RT treatment. We remain some distance from that objective. The peripheral blood of patients with cancers has yielded some markers of risk (Chaturvedi et al., 2010, Oh et al., 2011) and as such there is sound scientific reason to search for others that may indicate aspects of the response to radiotherapy.

Systematically collected samples with the use of a standard operating procedure in sufficient numbers for biomarker analysis are difficult to obtain. However, we have devised a system where a sufficiently powered study needs relatively few samples (Zhou et al., 2012). This allows a serious beginning to biomarker discovery where the Bayesian approach of accretion of information and testing can take us towards a panel of biomarkers. Therefore our proteomics pipeline can assist in developing personalised approaches to the treatment of lung cancer. Our specific approach relied on high end mass spectrometry with 8 channel isobaric tagging for relative quantification and the use of reference standard to allow intra experiment comparisons. Reproducible depletion of major protein constituents allows deeper penetration into the proteome, with low level constituents identified. Technical variation of the workflow is very low with selection of high confidence peptide spectral matches ensuring that quantification is only calculated from good quality matches, reducing technical variation and ensuring low false discovery rates. The largest variation present in the samples for these types of studies being inter person variation, a longitudinal approach with two baseline samples allows the variation for a specific protein to be assessed, thus reducing its impact. With a pre- and during-treatment sample the relative expression of a protein within a patient sample can be calculated allowing differences in an individual proteome to be monitored without being lost in the background inter-person biological variation (Zhou et al., 2012, Zhou et al., 2013).

One third of lung cancer cases present with locally advanced disease stage (stage III) and one third with stage IV disease (Morgensztern et al., 2010). The standard of care for locally advanced disease is concurrent chemo-radiotherapy (Auperin et al., 2010) but the majority are not suitable for this approach due to comorbidities and advanced age (De Ruysscher et al., 2009). An alternative treatment is sequential chemoradiotherapy. The patients included in this study were at least stage 3 and were being treated with sequential chemoradiotherapy and so are representative of a standard care population for this disease (Reck et al., 2013). The radiotherapy treatment was received by patients in our study over 4–6.5 weeks, with samples collected early during treatment. The biomarkers discovered have the potential to stratify patients into two groups, based on survival, at an early stage of treatment and possible help tailored treatment for patients in respect of radiotherapy. Studies on a prognostic model in NSCLC patients undergoing chemoradiotherapy treatment have previously been performed. This took into account five clinical variables (gender, performance status, forced expiratory volume, number of lymph node stations and tumour volume) (Dehing-Oberije et al., 2009), the performance of this model was improved upon the addition of two blood borne biomarkers CEA and IL-6 (Dehing-Oberije et al., 2011). These two markers were identified by subjective choice of candidate proteins and so it would be of interest to see if LRG1 and LBP levels during radiotherapy would add to this model.

We have presented data showing the discovery proteomic identification of three plasma proteins which are putative predictive marker of survival for SqCC NSCLC patients after radiotherapy. The level of two of these proteins (LRG1 and CRP) has been shown to be predictive with regard to survival, with elevation indicative of reduced survival time. A combined level of over 140 mg/L in plasma was found in all of the patients with shorter survival. The validation was carried out using a non-MS orthogonal antibody-based method, improving confidence that the differences seen are real and therefore, potentially of use in a clinical setting. The agreement, observed in this study and others, between isobaric tagging experiments and ELISA supports the use of an isobaric tagging approaches to the identification of novel biomarkers in bodily fluid before validation by other methods like ELISA.

One of the biomarkers we have highlighted, CRP, has previously been proposed as an agent allowing monitoring of chronic inflammation (reviewed Volanakis, 2001) and also been studied as a possible biomarker of lung cancer risk (Chaturvedi et al., 2010, Van Hemelrijck et al., 2011). Elevated levels of circulating CRP were associated with increased risk of lung cancer; elevated levels were observed up to 5 years pre-diagnosis. Since chronic inflammation has been proposed to generate an environment advantageous to cancer survival, as well as promoting tumourogenesis (NSCLC reviewed in O'Callaghan et al., 2010) the identification of CRP as a risk factor may be due to chronic inflammation. Thus, because of the non-specific nature of circulating CRP levels Hemelrijick and colleagues measured multiple time points of CRP and observed that this increased confidence in the link between elevation of CRP and lung cancer risk (Van Hemelrijck et al., 2011). Our study also allowed multiple reading of CRP, thus allowing the increase in this protein during radiotherapy treatment to be observed; therefore multiple time points should be implemented into any potential future studies. The levels of CRP which indicated risk of lung cancer in the study by Hemelrijick and colleagues was over 10 mg/mL. It is worth noting that the elevated levels we see during radiotherapy are higher (up to 80 mg/L), with the pre radiotherapy levels averaging 34 mg/mL. In NSCLC elevated levels of plasma CRP has been linked with poor prognosis for patients undergoing surgical resection and chemotherapy. But to our knowledge this is the only report of CRP elevation as a potential predictive marker for survival after radiotherapy. This indicates that CRP measurement may be useful for all lung cancer patients undergoing treatment. Prognosis for resection and chemotherapy were indicated by levels before treatment, where the link we have observed is a change during therapy. Therefore the mechanism for CRP release may differ between the different therapies and so more work needs to be carried out to investigate the mechanism of the CRP elevation. CRP as a predictive marker of survival after radiotherapy has been previously investigated by Dehing-Oberjie and colleagues as part of a nine protein panel; they did not see CRP levels as a significant indicator (Dehing-Oberije et al., 2011). However only a single time point prior to treatment was acquired. We see no significant difference in pre-treatment levels of CRP in this study which confirms their observation.

Lipopolysaccharide binding protein is another acute phase response protein, involved in the immune response to gram negative bacteria. It has been monitored to predict outcome for sepsis and lung injury (Villar et al., 2009) and a study has shown it may be of use for patients with neutropenia associated with cancer to diagnose those with gram negative infections (Oude Nijhuis et al., 2003). Modulation of this protein has not previously been linked with lung cancer or radiotherapy but it is known that it can be expressed by lung epithelial cells upon stimulation by cytokines like IL-6 (Dentener et al., 2000). Therefore the LBP increase seen during treatment could be as a result IL-6 stimulation of the lung epithelial rather than an immune challenge. Previous reports have shown that IL-6 can be expressed in lung cancer cell lines and the lungs of patients after radiotherapy (Zhang et al., 1994). Measurement of IL-6 in the plasma of the patients during radiotherapy in our study showed some correlation with the level of CRP and LRG1, but not LBP. Previously it has been reported that elevated IL-6 plasma levels prior to treatment have been associated with poor prognosis for radiotherapy patients (Dehing-Oberije et al., 2011). With our findings, more investigation is required into whether CRP and LRG1 levels alone can be independent predictors of survival or whether a panel including IL-6 would have greater predictive ability. The identification of CRP and LBP in this study and IL-6 previously (Dehing-Oberije et al., 2011) as indicators of response to radiotherapy suggests that inflammation could be an important factor in radiotherapy response either through tumour response or radiotherapy toxicity. IL-6 levels have previously been shown to be associated with lung toxicity (Siva et al., 2014) and so it would be interesting to see if CRP and LRG1 modulation in plasma could indicate radiotherapy linked toxicity.

The levels of LRG1 have been shown to be elevated in the serum and urinary exosomes of lung cancer patients (Li et al., 2011, Liu et al., 2012) with this protein also being identified in lung tumour tissue. The role of LRG1 is not fully understood, although it is of interest due to its role in angiogenesis; where it acts as a pro-angiogenic factor modulating the role of TGF-β (Wang et al., 2013). Wang and colleagues have also shown that angiogenesis can be reduced by the inhibition of LRG1 and so it is a possible therapeutic target for regulation of angiogenesis. It is known that during radiotherapy angiogenic factor expression is modulated with a correlative increase in angiogenesis (Sofia Vala et al., 2010, Gu et al., 2013). Therefore the finding that LRG1 is elevated in lung cancer patients with poor response to radiotherapy indicates it may have three clinical uses: as a therapeutic target to increase the efficacy of radiotherapy, as a tool to stratify patients who require angiogenesis inhibitors in combination with radiotherapy (Wang et al., 2013) and as shown in this study as a biomarker for poor prognosis.

## 5 Conclusion

We have identified two potential protein blood borne predictive markers for survival which could be used to stratify patients with squamous cell carcinoma early during radiotherapy. They have been verified on an additional independent cohort of patients using ELISA. However larger studies need to be carried out and further analyses should be done integrating standard clinical factors predictive for survival after RT such as extent of lymph node involvement and performance status. Finally such predictive models will need to be validated on external cohorts.

The following are the supplementary data related to this article

Supplementary Table 1 Discovery cohort clinical characteristics. Male (M) Female (F).

Supplementary Table 2 Verification cohort clinical characteristics. Mean ± SD < 14 mo n = 11, > 18 mo n=12.

Supplementary Table 3 Number of peptide and protein identifications in each iTRAQ experiment. All datasets had a false discovery rate less than 0.5%. PSM (peptide spectral matches).

Supplementary Table 4 All proteins quantified using global discovery proteomics. Quantification is given as log2 ratio of during:before radiation for each patient individually. Patients 1–3 are survival < 14 mo and 4–6 survival > 18 mo.

Supplementary Table 5 All proteins identified using global discovery proteomics. All protein identified in each iTRAQ experiment independently with numbers of peptides and % coverage.

Supplementary Table 6 All peptides identified and quantified using global discovery proteomics. Quantification is given as area of iTRAQ region for each peptide spectral match. Any non-tryptic cleavages and missed cleavages are shown under cleavage.

Supplementary Table 7 All peptides identified and quantified using global discovery proteomics. Quantification is given as area of iTRAQ region for each peptide spectral match. Any non-tryptic cleavages and missed cleavages are shown under cleavage.

Supplementary Table 8 All peptides identified and quantified using global discovery proteomics. Quantification is given as area of iTRAQ region for each peptide spectral match. Any non-tryptic cleavages and missed cleavages are shown under cleavage.

Supplementary Document 1 Proteome viewer help section. Description of how to access and view the proteomic dataset on public website http://www.scalpl.org/hank/MatchPage;jsessionid=80bf3253ea31befd6e36c2ce957a?0.

Supplementary Fig. 1 Calculation of within-person variation. Histogram of protein changes between the two baselines was demonstrated in the figure, indicating that the within-person variation generally follows a Normal distribution with extreme values. Further investigation showed that a large proportion of the extreme protein changes were due to technical variation (data not shown).

## Role of Funding Sources

This work was supported by the Cancer Research UK Experimental Cancer Medicine Centre code R114689 A07, and Swedish Cancer Society CAN2010/1335 and Leukemia Lymphoma Research code 13005. No funding source was involved in the writing of this manuscript or the decision to submit it for publication.

## Author Contributions

MJW and ADW wrote the manuscript and reviewed all data and conceived the study with CD. LJCP, CFF, FHB, and PK provided clinical material, helped design the study and reviewed the manuscript. MJW, AJKW, MP, and AB performed the experiments. MJW, CZ, MP, and AJKW performed informatics analyses.

## Conflicts of Interest

The authors declare no conflict of interest.

## Figures and Tables

**Table 1:** Discovery proteomics data for proteins which change in the plasma of patients during radiotherapy. Table includes proteins with a significant change (p value < 0.05) for either change after radiotherapy (independent of outcome) or significantly different between survival groups (p value < 0.05). Ratios are the average of all patients in group. All the p-values shown in the table have been corrected for multiple comparisons.

Accession number	Gene symbol	Number of peptides	Average log2 change during radiotherapy — all patients	P value (for average change of all patients)	Average log2 change during radiotherapy — < 14 mo	Average log2 change during radiotherapy — > 18 mo	P value (for difference between patient cohorts)
ENSP00000232003	HRG	472	− 0.29	0.03	− 0.35	− 0.25	0.99
ENSP00000364494	APOC3	97	− 0.68	0.06	− 1.21	− 0.17	0.001
ENSP00000441450	LBP	33	0.35	0.05	0.63	0.12	0.42
ENSP00000252491	APOC1	35	− 0.37	0.25	− 0.85	0.07	0.005
ENSP00000246662	KRT9	92	0.14	0.62	0.65	− 0.26	0.005
ENSP00000310861	KRT2	41	− 0.01	0.76	0.78	− 0.79	0.01
ENSP00000255030	CRP	63	1.13	0.20	2.20	0.06	0.01
ENSP00000302621	LRG1	334	0.34	0.18	0.68	0.02	0.02
ENSP00000252490	APOC2	45	− 0.28	0.53	− 0.88	0.30	0.05

**Table 2:** Protein idenfied from the acute phase response. Table includes acute phase response proteins identifed in investigation with expected change of direction with activation of the acute phase response. P value indicates significant difference between < 14 mo and > 18 mo survival patient groups.

Protein (gene symbol)	P value	Observed direction of change during radiotherapy	Expected direction of change during radiotherapy
CRP	0.01	↑	↑
LBP	0.42	↑	↑
F2	0.97		↑
HPX	0.99		↑
VWF	0.99		↑
CP	0.73		↑
PLG	0.99		↑
ITIH4	0.05		↑
APCS	0.72		↑
C2	0.99		↑
SerpinA3	0.05		↑
C5	0.80		↑
SerpinF	0.99		↑
FGA	0.42		↑
SerpinD1	1.0		↑
C4BP	0.97		↑
A2M	0.91		↑
AGT	0.92		↑
C3	0.92		↑
C9	0.44		↑
AHSG	0.73		↓
TTR	0.64		↓
ApoH	0.96		↓
ApoA2	0.78		↓
AMBP	0.94		↓
HRG	0.96		↓
