# Supplementary material for: Discovery and Validation of Predictive Biomarkers of Survival for Non-small Cell Lung Cancer Patients Undergoing Radical Radiotherapy: Two Proteins With Predictive Value
Source: eBioMedicine. 2015 Jun 19;2(8):841–50. doi: 10.1016/j.ebiom.2015.06.013 (PMC4563120; doi:10.1016/j.ebiom.2015.06.013)
Supplement: Supplementary Table 1 — Discovery cohort clinical characteristics. Male (M) Female (F). [file mmc1.docx]

**Supplementray Table 1. Discovery cohort Clinical characterisitics.** Male (M) Female (F).

| Patient | Weight (kg) | Gender | Weight Loss | Performance status | TNM Staging | Survival (days) | Smoking Status | Pack years |
| --- | --- | --- | --- | --- | --- | --- | --- | --- |
| 1 | 65 | F | <5% | 1 | 3B | 399 | Ex-smoker | 6 |
| 2 | 83 | F | None | 1 | 3 | 356 | Ex-smoker | 32 |
| 3 | 73 | F | >10% | 1 | 3B | 204 | Ex-smoker | 45 |
| 4 | 93 | M | None | 1 | 3B | 756 | Ex-smoker | 36 |
| 5 | 81 | M | None | 0 | 3 | 539 | Ex-smoker | 14 |
| 6 | 79 | M | <5% | 1 | 3B | 644 | Ex-smoker | 84 |
