# Supplementary material for: Discovery and Validation of Predictive Biomarkers of Survival for Non-small Cell Lung Cancer Patients Undergoing Radical Radiotherapy: Two Proteins With Predictive Value
Source: eBioMedicine. 2015 Jun 19;2(8):841–50. doi: 10.1016/j.ebiom.2015.06.013 (PMC4563120; doi:10.1016/j.ebiom.2015.06.013)
Supplement: Supplementary Table 2 — Verification cohort clinical characteristics. Mean ± SD < 14 mo n = 11, > 18 mo n=12. [file mmc2.docx]

**Supplementary Table 2. Verification cohort Clinical chracterisitcs.** Mean±SD<14 months n=11, >18 months n=12.

|  | | <14 months | >18 months |
| --- | --- | --- | --- |
| Number of patients | | 11 | 12 |
| Weight (Kg) | | 69±16 | 68±13 |
| Gender | Male | 6 | 5 |
|  | Female | 5 | 7 |
| Histology | Squamous cell carcinoma | 5 | 6 |
|  | Adenocarcinoma | 3 | 3 |
|  | Small cell | 3 | 3 |
| Weight Loss | None | 2 | 5 |
|  | <5 % | 2 | 4 |
|  | 5-10% | 4 | 1 |
|  | >10% | 3 | 2 |
| Performance Status | 0 | 2 | 0 |
|  | 1 | 7 | 11 |
|  | 2 | 2 | 1 |
| Staging (TNM) | 3 | 4 | 4 |
|  | 3A | 3 | 2 |
|  | 3B | 4 | 5 |
|  | 3/4 | 0 | 1 |
| Smoking status | Current Smoker | 2 | 3 |
|  | Ex smoker | 9 | 8 |
|  | Never smoked | 0 | 1 |
| Pack years | | 52±34 | 39±28 |
