# Supplementary material for: Discovery and Validation of Predictive Biomarkers of Survival for Non-small Cell Lung Cancer Patients Undergoing Radical Radiotherapy: Two Proteins With Predictive Value
Source: eBioMedicine. 2015 Jun 19;2(8):841–50. doi: 10.1016/j.ebiom.2015.06.013 (PMC4563120; doi:10.1016/j.ebiom.2015.06.013)
Supplement: Supplementary Table 3 — Number of peptide and protein identifications in each iTRAQ experiment. All datasets had a false discovery rate less than 0.5%. PSM (peptide spectral matches). [file mmc3.docx]

**Supplementary Table 3. Number of peptide and protein identifications in each iTRAQ experiment.** All datasets had a false discovery rate less then 0.5%. PSM (peptide spectral matches).

|  | Experiment 1 | Experiment 2 | Experiment 3 |
| --- | --- | --- | --- |
| Number of PSM | 159352 | 229064 | 234955 |
| Number of peptides | 10343 | 22050 | 25493 |
| Number of proteins | 306 | 458 | 339 |
