# Supplementary material for: Discovery and Validation of Predictive Biomarkers of Survival for Non-small Cell Lung Cancer Patients Undergoing Radical Radiotherapy: Two Proteins With Predictive Value
Source: eBioMedicine. 2015 Jun 19;2(8):841–50. doi: 10.1016/j.ebiom.2015.06.013 (PMC4563120; doi:10.1016/j.ebiom.2015.06.013)
Supplement: Supplementary Document 1 — Proteome viewer help section. Description of how to access and view the proteomic dataset on public website http://www.scalpl.org/hank/MatchPage;jsessionid=80bf3253ea31befd6e36c2ce957a?0. [file mmc9.docx]

Proteome Viewer help section

Global protein analysis

Protein level quantification is viewable in a heat map, each hexagon represents a different protein group, with red indicating a rise and green a reduction. A significant change for a channel can be shown by clicking on the label on the box to the right. This will highlight with a yellow edging the protein that are changing in that condition. By clicking on the protein of interest on the map the protein information is shown.


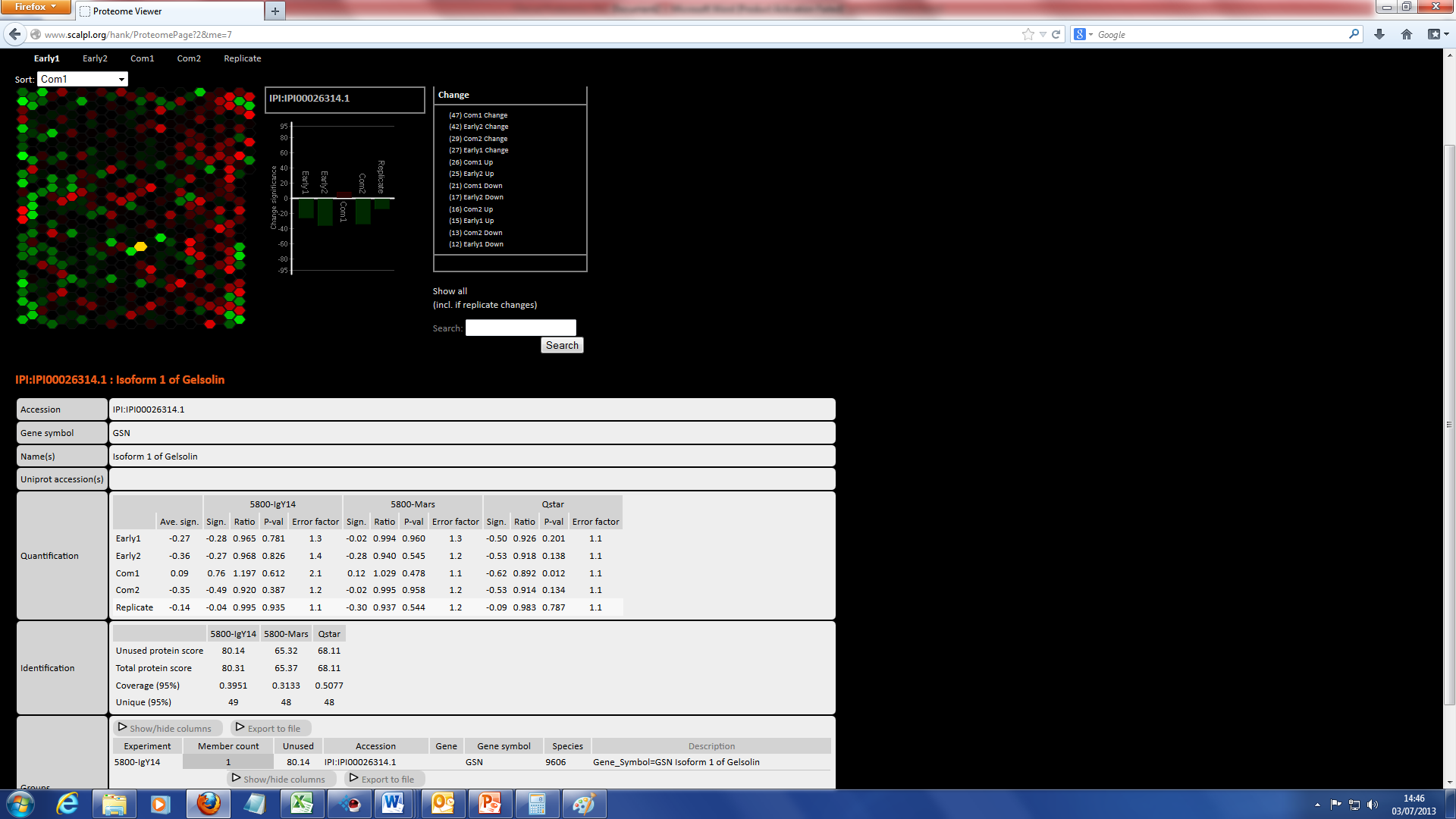


Figure 1. Screen shot of protein level data with search box and match count circled for peptide level data.

Specific Protein Information

Specific proteins can be searched for using the search box at the bottom right. This will bring up the protein level data. Peptide information and spectra can be reached by clicking on member count in the group section. This process is slow, but it will show the peptides that were used to identify the protein. Clicking then on match count, followed by spectrum will show you the areas used for the quantification. Finally identifier will show the spectra used for identification of that peptide spectral match.


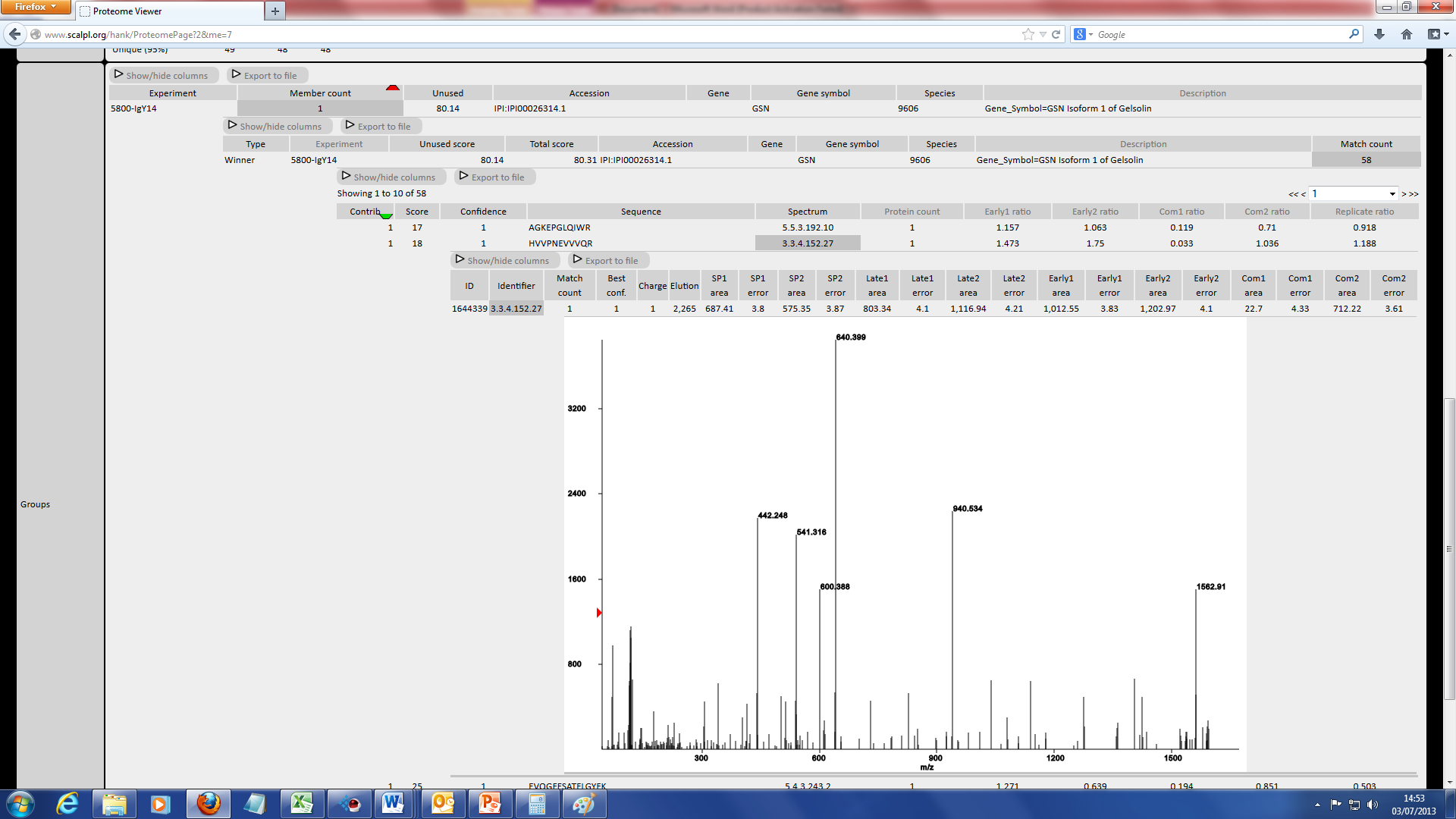


Figure 2. Screen shot of peptide level data with the route required to see peptide spectra circled in red.
