# Supplementary figures and images for: Discovery and Validation of Predictive Biomarkers of Survival for Non-small Cell Lung Cancer Patients Undergoing Radical Radiotherapy: Two Proteins With Predictive Value
Source: eBioMedicine. 2015 Jun 19;2(8):841–50. doi: 10.1016/j.ebiom.2015.06.013 (PMC4563120; doi:10.1016/j.ebiom.2015.06.013)

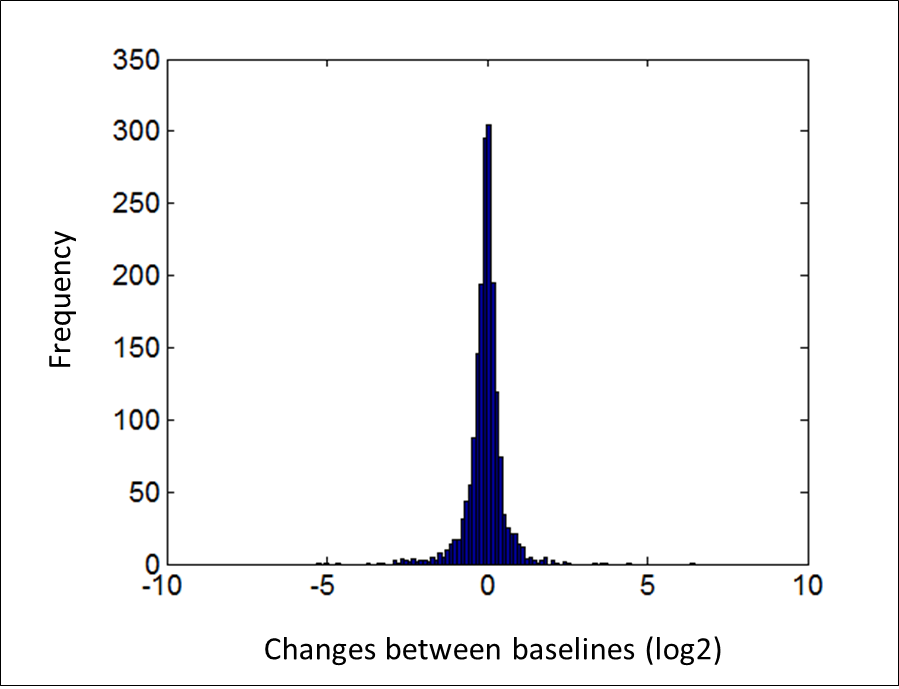

Supplement: Supplementary Fig. 1 — Calculation of within-person variation. Histogram of protein changes between the two baselines was demonstrated in the figure, indicating that the within-person variation generally follows a Normal distribution with extreme values. Further investigation showed that a large proportion of the extreme protein changes were due to technical variation (data not shown). [file mmc10.zip › Suplementary figure 1.png]
